# Impact of pharmacist-physician collaboration on patient outcomes in Parkinson’s disease: a randomised controlled trial in tertiary care

**DOI:** 10.1007/s11096-025-01883-6

**Published:** 2025-02-13

**Authors:** Phanutgorn Techa-angkoon, Yuvadee Pitakpatapee, Weerawat Saengphatrachai, Prachaya Srivanitchapoom, Thanarat Suansanae

**Affiliations:** 1 Department of Pharmacy, Faculty of Pharmacy, Mahidol University, 447 Sri Ayutthaya Road, Ratchathewi, Bangkok, 10400 Thailand; 2 Division of Neurology, Department of Medicine, Faculty of Medicine Siriraj Hospital, Mahidol University, Bangkok, 10700, Thailand

**Keywords:** Drug-related problems, Parkinson’s disease, Pharmacist, Pharmaceutical care

## Abstract

**Background:**

Previous studies have shown that reducing drug-related problems (DRPs) may improve therapeutic outcomes in patients with Parkinson’s disease (PD).

**Aim:**

To investigate the impact of pharmacist participation in Parkinson’s disease clinic on the number of DRPs, clinical outcomes, and the quality of life of PD patients.

**Method:**

This single-blinded randomised controlled trial was conducted at the Parkinson’s Disease and Movement Disorders Clinic. Patients aged ≥ 18 years, diagnosed with idiopathic PD for at least 3 years, and receiving antiparkinsonian drugs were randomly assigned (1:1) to the pharmacist-physician (PP) or usual care (UC) groups. The primary outcome was changes in the number of DRPs from baseline to 24 weeks between groups. Secondary outcomes included the Movement Disorder Society-Sponsored Revision of the Unified Parkinson’s Disease Rating Scale (MDS-UPDRS) score, eight-item version of the Parkinson’s Disease Questionnaire (PDQ-8) score, and Patients’ Global Impression of Change (PGIC) score at week 24.

**Results:**

A total of 80 patients were randomised, with 40 in each group. The mean number of DRPs reduced in both groups; however, the reduction was greater in the PP group compared to the UC group (− 7.2 ± 3.6 vs. − 3.0 ± 1.8, *p* < 0.001), especially non-adherence issues. The MDS-UPDRS and PDQ-8 scores showed significantly greater improvement in the PP group. A higher proportion of patients in the PP group achieved improvement in PGIC scales compared to those in the UC group.

**Conclusion:**

Our findings demonstrated that pharmacist-physician collaboration service in the PD clinic positively impacted patient outcomes.

**Trial registration:**

ClinicalTrials.gov NCT05410210 (date 13 May 2022).

**Supplementary Information:**

The online version contains supplementary material available at 10.1007/s11096-025-01883-6.

## Impact statements

- Pharmacist participation in a multidisciplinary healthcare team can reduce DRPs in patients with PD and increase the opportunities to achieve the PD therapeutic goals.
- Non-adherence is the main DRP in patients with PD which can be resolved. Therefore, healthcare professionals should regularly monitor their medication adherence and manage each patient individually.

## Introduction

Parkinson’s disease (PD) is a progressive, neurodegenerative disease involving the loss of dopaminergic neurons in the substantia nigra pars compacta. PD is also associated with motor and non-motor symptoms. When PD progresses, symptoms become more complex as the disease advances, and long-term complications of treatment such as motor and non-motor fluctuations, dyskinesia, and psychosis [1]. These symptoms lead to a decline in functional abilities and quality of life (QoL) [2, 3]. Thus, PD therapy aims to minimize symptoms and improve QoL [4]. Currently, the mainstay for PD management is still medication [1]. PD therapy commonly involves prescribing drugs that act on dopamine. These medications require multiple daily doses in complex regimens, particularly levodopa [5]. Moreover, most PD patients are elderly, have comorbidities, and are concurrently taking various medications [6]. Therefore, PD patients are more likely to experience drug-related problems (DRPs), especially non-adherence [5–7].

The DRPs decrease the likelihood of achieving the desired PD therapeutic outcomes. Previous studies demonstrating a reduction in DRPs seem likely to improve therapeutic outcomes in PD patients [7]. Most pharmacist interventions in earlier studies included medication review, DRPs assessment, adherence assessment, or patient education [8]. Several studies have previously demonstrated the beneficial effects of pharmaceutical care on PD patient outcomes, which tended to decrease the number of DRPs [6, 7, 9], and improve adherence [7, 10], symptoms [11], and QoL [10, 12]. However, some studies have shown different results in clinical outcomes and QoL [7, 13]. To date, no trial has confirmed the benefits of multifaceted pharmaceutical care involving a multidisciplinary team in a PD clinic in outpatient tertiary care settings. Therefore, we designed this randomised controlled trial to evaluate the benefits of pharmacist-physician collaboration from the perspective of DRPs, clinical outcomes, QoL, and overall health status in PD patients.

### Aim

To investigate the impact of pharmacist participation in the Parkinson’s Disease and Movement Disorders Clinic on the number of DRPs, clinical outcomes, and QoL in PD patients.

### Ethics approval

This study was approved by the Siriraj Institutional Review Board (MU-MOU COA166/2022, 10/02/2022), and registered on ClinicalTrials.gov (NCT05410210). Written informed consent was obtained from all participants included in the study prior to randomisation.

## Method

### Study design and setting

This single-center, single-blinded, randomised controlled trial was conducted at the outpatient Parkinson’s Disease and Movement Disorders Clinic, Siriraj Hospital, Faculty of Medicine Siriraj Hospital, Mahidol University, Thailand from March 2022 to December 2022. Study visits were conducted at weeks 0, 12, and 24.

### Participants

The inclusion criteria included: (1) ≥ 18 years old; (2) diagnosed with idiopathic PD according to the United Kingdom Parkinson’s Disease Society (UKPDS) Brain Bank Criteria for at least 3 years; (3) modified Hoehn and Yahr stage 2–4; (4) received at least one of antiparkinsonian drugs; and (5) has given a written informed consent to participate in the study. The exclusion criteria included: (1) unable to communicate via phone or internet, (2) terminal illness; (3) bedridden; (4) history of other movement disorders (corticobasal syndrome, multiple system atrophy, progressive supranuclear palsy, or essential tremor); (5) moderate to severe dementia as evidenced by Thai Mental State Examination (TMSE) less than 20 and without the caregiver; (6) planning to undergo surgery, or having a history of surgical treatment for PD; (7) hearing impairments; or (8) unable to communicate in Thai.

Patients who consented but later declined, missed pharmaceutical care appointments, needed earlier treatment, or were in the usual care group with clinically significant DRPs were eligible for withdrawal. The criteria were assessed through medical records and patient interviews.

### Randomisation

All eligible patients were randomly assigned into one of the two groups, either the pharmacist-physician (PP) group or the usual care (UC) group in a 1:1 ratio using a computerized block randomisation sequence with a block size of 4. The results were sealed in sequentially numbered envelopes and prepared by an independent person not associated with this study. These envelopes were subsequently opened by a pharmacist (PT) after obtaining the patient’s informed consent at the clinic. Participants and the Movement Disorder Society-Sponsored Revision of the Unified Parkinson’s Disease Rating Scale (MDS-UPDRS) assessors were blinded.

### Data collection and Interventions

**Fig. 1:**
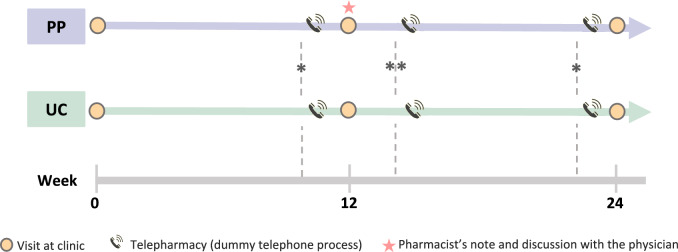
Study schematic. *The process before weeks 12 and 24 consists of reminding patients about their appointments, reminding them to bring their medications, and detecting drug-related issues. The UC group is only involved in the first two activities. **Within one week after week 12, the operational process includes verifying the medication list against the prescription, providing patient education, and offering medication counseling. The UC group only performs the first process. PP = pharmacist-physician group, UC = usual care group

At week 0, the pharmacists collected all patients’ baseline data from medical records and face-to-face interviews (Fig. 1). Using this information, pharmacists identified DRPs using the study’s structured case record form. Both groups received usual care at the PD outpatient clinic, led by a healthcare professional team (neurology residents, fellows, neurologists, and nurses), and obtained medications at the pharmacy department.

One-week before the 12-week telephone calls were made to remind them regarding their appointments, ensure they brought their medications to the clinic, and conduct a medication history to identify DRPs. In the UC group, only the first two tasks were performed.

At the 12-week visit, pharmacists will identify and assess DRPs again, compiling all identified DRPs as the baseline DRPs. The pharmacist responsible for the intervention had specialized knowledge in the management of PD. For the PP group, patients will undergo medication reconciliation and medication review. DRPs and recommendations for resolution will be reported to physicians using a structured form (Supplementary material 1). Subsequently, pharmacists and physicians will collaborate to decide on appropriate interventions for the patients.

One-week after 12-week visit, the pharmacist contacted both groups to verify the medication list against the prescription to ensure completeness. In the PP group, the pharmacist also provided education on PD, drug information, the importance of medication adherence, and lifestyle modifications tailored to individual needs (Supplementary material 2).

### Outcomes and measurements

The primary outcome was the change in the number of total DRPs at week 24 from baseline between groups. The secondary outcomes were the change at week 24 and within groups in the number of DRPs related to PD treatment, the MDS-UPDRS scores (both total score and each part) [14], the 8-item PD questionnaire (PDQ-8) scores [15]. Proportion of patients who achieved an improvement in Patient Global Impression of Change (PGIC) scales [16] at 24 weeks was also assessed as another secondary outcome. Assessment of DRPs was carried out by two clinical pharmacists (PT and TS) at weeks 0, 12, and 24. All identified DRPs were initially evaluated by PT and were re-evaluated and confirmed by TS who was blinded to group assignment. The DRPs were classified according to Cipolle et al. [17], as the following: non-adherence, adverse drug reactions (ADRs), needs additional drug therapy, dosage too low, dosage too high, unnecessary drug therapy, and ineffective drug (Supplementary material 3).

The MDS-UPDRS assessed various aspects of PD symptoms. It consists of four parts: Part I explores non-motor experiences of daily living, Part II assesses motor experiences of daily living, Part III comprises motor examination by clinician, and Part IV evaluates motor complications [14]. The MDS-UPDRS was evaluated by blinded assessors in the ON state at the clinic. The PDQ-8 was used to represent the patient’s QoL [15]. Patients/caregivers completed the PDQ-8 at the clinic. The PGIC scale assessed overall changes in a patient’s health status since the beginning of treatment [16], and it was also completed by patients or caregivers at the clinic. The MDS-UPDRS, PDQ-8, and PGIC were used with permission from their developers.

### Sample size

The sample size calculation was based on the study of Foppa et al. [7], pharmacist interventions were shown to significantly reduce DRPs from 1.7 to 0.8 problems per patient. To detect a difference in the primary outcome with a power of 90% and an alpha of 0.05, the study required 33 patients in each group. To account for potential drop-outs, we adjusted the sample size by 20%, resulting in an actual sample size of 40 patients per group.

### Statistical analysis

Data were analyzed by using SPSS v.18.0 software. All analyses were performed on an intention-to-treat (ITT). Missing data of the ITT population were estimated using the last observation carried forward method. The Shapiro–Wilk analysis is used to examine the normal distribution of the data. The differences between the two groups were analyzed using independent t-test or the Mann–Whitney U test for continuous variables and the Chi-square test for categorical variables. The differences between baseline and week 24 in each group were analyzed using paired *t*-test or the Wilcoxon rank sum test, as appropriate. A *P*-value < 0.05 was considered statistically significant.

## Results

**Fig. 2:**
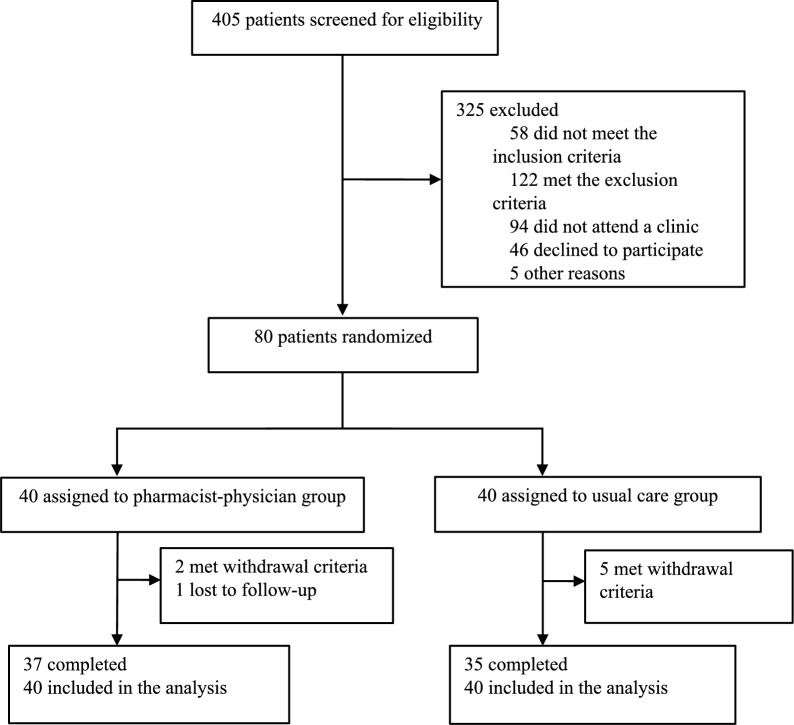
Flow diagram of participation in the study

A flowchart of participants throughout the study is shown in Fig. 2. A total of 80 patients were enrolled in the study; 40 were randomly assigned to the PP group and 40 to the UC group. All randomised patients were included in the analyses of both primary and secondary outcomes. The patient characteristics are presented in Table 1. Both groups exhibited similar baseline characteristics, except for PD duration (*p* = 0.012). Baseline MDS-UPDRS total scores and part scores did not differ significantly between groups.

**Table 1:** Patient characteristics in the pharmacist-physician and the usual care groups

Characteristics	PP (n = 40)	UC (n = 40)	*P* value^a^
Male, n (%)	22 (55.0)	22 (55.0)	1.000
Age, mean (SD), y	67.5 (11.4)	68.4 (9.7)	0.713
Marital status, n (%)			0.340
Single/Widowed/Separated	15 (37.5)	11 (27.5)	
Married	25 (62.5)	29 (72.5)	
Education, n (%)			0.104
≤ 12 years	22 (55.0)	29 (72.5)	
> 12 years	18 (45.0)	11 (27.5)	
Alcohol, n (%)			0.822
Never	30 (75.0)	30 (75.0)	
Past	8 (20.0)	9 (22.5)	
Present	2 (5.0)	1 (2.5)	
Smoking, n (%)			0.152
Never	30 (75.0)	35 (87.5)	
Past	10 (25.0)	5 (12.5)	
Present	0	0	
Herb or dietary supplement, n (%)	9 (25.0)	10 (22.5)	0.793
PD duration, median (IQR), y^b^	5.0 (3.5–8.5)	7.5 (5.0–11.5)	0.012
Levodopa duration, n (%)			0.073
≤ 5	25 (62.5)	17 (42.5)	
> 5	15 (37.5)	23 (57.5)	
H&Y stage, n (%)			0.364
2	24 (60.0)	18 (45.0)	
2.5	7 (17.5)	10 (25.0)	
3	8 (20.0)	8 (20.0)	
4	1 (2.5)	4 (10.0)	
Caregiver, n (%)	8 (20.0)	16 (40.0)	0.051
Number of all medications, median (IQR), n^b^	9.0 (5.5–11.0)	9.0 (6.0–13.0)	0.499
Number of medications for PD, n (%)			
Levodopa	38 (95.0)	40 (100)	0.152
Dopamine agonists	19 (47.5)	24 (60.0)	0.262
MAO-B inhibitors	7 (17.5)	8 (20.0)	0.775
COMT inhibitors	15 (37.5)	18 (45.0)	0.496
Trihexyphenidyl	5 (12.5)	2 (5.0)	0.235
LEDD, median (IQR), mg^b^	585 (362–906)	647 (425–932)	0.510
Levodopa daily dose, median (IQR), mg^b^	575 (300–750)	525 (400–800)	0.579
Levodopa daily dose, n (%)			0.816
≤ 600 mg	26 (65.0)	25 (62.5)	
> 600 mg	14 (35.0)	15 (37.5)	
Drug-related problems, mean (SD)			
Total DRPs/person	10.7 (4.0)	11.9 (3.9)	0.172
PD treatment-related DRPs/person	8.2 (3.6)	9.7 (4.1)	0.087
MDS-UPDRS, median (IQR), scores^b^			
Total	48.0 (38.0–65.5)	58.5 (45.0–79.0)	0.071
Part I	8.0 (5.5–10.0)	8.0 (5.0–12.0)	0.721
Part II	10.0 (7.5–16.0)	15.5 (10.0–19.0)	0.065
Part III	27.5 (18.0–33.5)	31.0 (21.0–48.0)	0.122
Part IV	3.5 (1.5–6.5)	3.0 (2.0–5.5)	0.720
PDQ-8, mean (SD), scores	9.1 (5.6)	8.8 (5.8)	0.785

PD, Parkinson’s disease; H&Y, Hoehn & Yahr stage; MAO-B inhibitors, monoamine oxidase B inhibitors; COMT inhibitors, Catechol-O-methyl transferase inhibitors; LEDD, levodopa equivalent daily dose

^a^Independent t test was used for continuous variables and the Chi-square test for categorical variables

^b^Mann–Whitney U test

### Primary outcomes

**Fig. 3:**
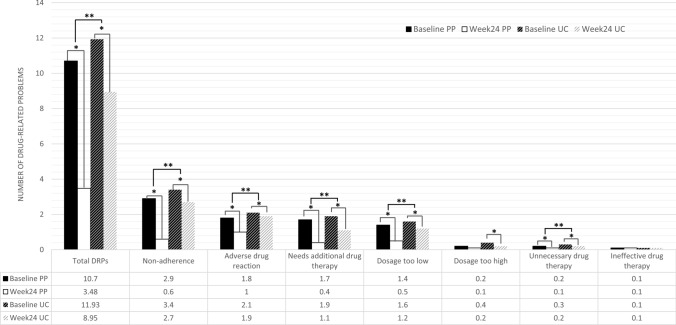
Changes from baseline in the number of total DRPs and each type of DRPs at 24 weeks. *p < 0.05 compared to baseline; **p < 0.05 when compared between groups

A total of 906 DRPs were identified at baseline, with 428 DRPs (10.7 per patient) in the PP group and 478 DRPs (11.9 per patient) in the UC group (Table 1). The number of DRPs was the cumulative count of each specific problem detected, with the same type of problem across different medications being counted cumulatively. The two groups had no significant difference in the mean number of DRPs (*p* = 0.172). At week 24, the PP group showed a greater reduction in total DRPs compared to the UC group (− 7.2 vs. − 3.0 from baseline; *p* < 0.001) (Fig. 3). Both groups showed significant reductions in total DRPs at week 24 compared to baseline (*p* < 0.001).

The most frequent types of DRPs at baseline were non-adherence (35%), followed by ADRs (19%), needs additional drug therapy (18%), dosage too low (14%), unnecessary drug therapy (9%), dosage too high (4%), and ineffective drug therapy (1%). At week 24, the PP group demonstrated a significant reduction in DRPs compared to UC group in the following categories: non-adherence (*p* < 0.001), ADRs (*p* = 0.001), needs additional drug therapy (*p* = 0.045), dosage too low (*p* = 0.005), and unnecessary drug therapy (*p* = 0.010). Interventions to resolve DRPs in the PP group are summarized in Table 2.

**Table 2:** Interventions related DRPs in the pharmacist-physician group^*^

DRPs at baseline	n	Pharmacist-physician interventions						Pharmacist-patient/caregiver intervention
		Addition of a new drug	Dose adjustment	Drug monitoring	Drug discontinuation	Rescheduling	Drug switching	Drug counseling
Non-adherence	152	–	3	7	12	6	1	123
Not taking medication as prescribed by a physician	70	–	2	3	7	4	–	54
Not using medication as recommended on the package insert	32	–	–	2	–	–	–	30
Forget to take medication	22	–	–	–	–	1	–	21
Self-adjusting the dosage regimen	14	–	1	–	1	1	–	11
Stop taking medication	14	–	–	2	4	–	1	7
Adverse drug reaction	79	3	15	42	7	4	1	7
Needs additional drug therapy	79	74	–	2	–	1	–	2
Dosage too low	62	2	40	10	3	7	–	–
Dosage too high	10	–	10	–	–	–	–	–
Unnecessary drug therapy	38	–	1	3	21	–	–	13
Ineffective drug	8	–	–	1	2	–	5	–
Total	428	79	69	65	45	18	7	145

*There might be some discrepancies in the method to solve the patient’s problem. The actual interventions were based on the mutual agreement between the pharmacist and physician

### Secondary outcomes

The reduction in the number of DRPs related to PD treatment at 24 weeks was greater in the PP group than in the UC group, in line with the primary outcome. Compared to the UC group, the PP group demonstrated a significant decrease in three types of DRPs—non-adherence, ADRs, and dosage too low.

**Fig. 4:**
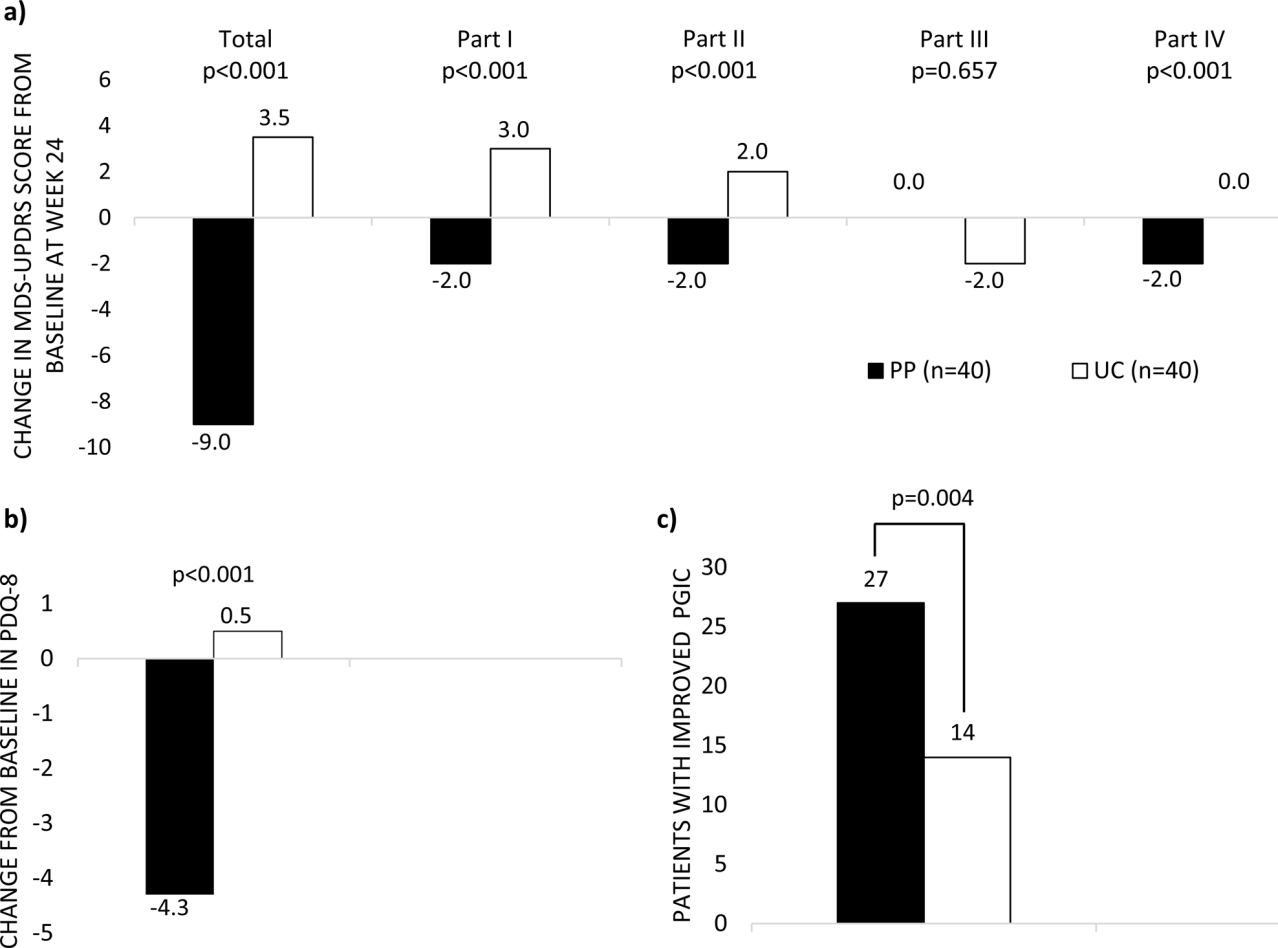
Change from baseline in **a** MDS-UPDRS scores, **b** PDQ-8 scores at week 24, and **c** Proportion of patients with improved overall health status at 24 weeks

At week 24, the improvement from baseline in MDS-UPDRS total, parts I, II, and IV scores was significantly greater in the PP group than in the UC group, except for part III (Fig. 4a). Within-group analysis revealed significant reductions in MDS-UPDRS total, parts I, II, and IV scores at 24 weeks in the PP group. Conversely, the UC group experienced significant increases in total, parts I, and II scores, with no significant change in part IV scores. Part III scores at week 24 did not differ from baseline in either group. Analysis of subitems of MDS-UPDRS part IV showed pharmacist-physician collaboration significantly improved time spent with dyskinesia (− 0.2 vs. 0.2; *p* = 0.007), time spent in the OFF state (− 0.8 vs. − 0.2; *p* = 0.001), functional impact of fluctuations (− 0.6 vs. 0.2; *p* = 0.005), and complexity of motor fluctuations (− 0.2 vs. 0.5; *p* = 0.005) compared to usual care at week 24. However, significant differences in the complexity of motor fluctuations between groups resulted from the worsening of the UC group. Moreover, there was a greater reduction in OFF-time duration at 24 weeks from baseline in the PP group compared to the UC group (median − 15.0 vs. 0.0 min, respectively; *p* = 0.001).

The pharmacist-physician collaboration significantly improved PDQ-8 scores compared to usual care at week 24 (Fig. 4b), especially in mobility (*p* < 0.001), communication (*p* = 0.002), bodily discomfort (*p* = 0.004), and stigma (*p* = 0.009). Analysis of PGIC showed that patients experienced an improvement (very much, much, or minimally) in overall health status during the study. Patients in the PP group significantly achieved an improvement in PGIC scales more than those in the UC group at 24 weeks (Fig. 4c).

## Discussion

### Statement of key findings

This randomised, controlled trial demonstrated that the participation of pharmacist in the Parkinson’s disease and Movement Disorders clinic had positive effects on the number of DRPs, especially in non-adherence, by week 24. Additionally, this study showed improvements in clinical outcomes, specifically regarding motor complications, QoL, and overall health status among patients with PD.

### Strengths and weaknesses

To our knowledge, this is the first randomised, controlled trial demonstrating improved DRPs outcomes in patients with PD through pharmacist-physician collaboration service in PD clinic. However, this study has some limitations. Firstly, we did not blind DRPs assessors and interventionists. Nevertheless, DRPs assessment was systematically evaluated using two independent assessors. Moreover, blinding the pharmacist’s intervention with the physician was impractical, allowing physicians to gain insights that could benefit other patients or lead to enhanced patient care. Secondly, the study only included PD patients at H&Y stages 2–4, excluding those with moderate to severe dementia or those planning or having undergone surgical PD treatment. Therefore, the findings could not apply to these populations. Lastly, as the study involved pharmacists who have experienced in neurology or PD clinic, the results may not be generalizable to settings without such experienced clinical pharmacists or well-trained pharmacists.

### Interpretation

Baseline characteristics in our study were similar in both groups, except for PD duration. Despite random assignment, the UC group exhibited a longer disease duration than the PP group, potentially influencing outcomes, especially regarding motor complications. According to a previous study in Chinese PD patients, disease duration is found to be a risk factor for motor fluctuation and dyskinesia [18]. Although, there were no differences in motor complications as indicated by MDS-UPDRS part IV and levodopa daily dose between the two groups at baseline in this study.

Non-adherence, particularly not taking medications as prescribed, was the most common DRPs in our study, consistent with prior research [7]. Patients with insufficient PD knowledge tended to exhibit lower medication adherence [19], highlighting the need for comprehensive education for both patients and caregivers. Our study found that pharmacist interventions led to the most noticeable improvements in non-adherence compared to other DRPs. This outcome could be attributed to the interventions, which involved counseling, educating patients about their diseases and prescribed medications, encouraging medication adherence, and adjusting regimens to individual needs. These interventions were consistent with suggestions highlighting the need for increased awareness of the consequences of non-adherence, along with collaboration with healthcare professionals and patients, to enhance medication adherence [20].

The study revealed significant reductions not only in non-adherence issues but also in other DRPs through pharmacist-physician collaboration compared to usual care. This reduction is attributed to the interventions, which were jointly implemented by healthcare professionals with appropriate frequency, thereby enhancing their efficacy in managing DRPs (Table 2). Moreover, the involvement of pharmacists who have experienced in neurology or PD clinic in the multidisciplinary teams plays a pivotal role in addressing complex DRPs. For example, a pharmacist could identify nocturnal hypokinesia which was a cause of patient’s insomnia and recommended adding controlled-release levodopa/benserazide at bedtime. The symptoms of insomnia and nocturnal hypokinesia had been resolved after this adjustment.

Considering clinical outcomes, the PP group showed a significant reduction in MDS-UPDRS scores compared to baseline, consistent with previous findings [11] on the effectiveness of pharmaceutical care in lowering scores in PD patients. However, our study has too longer follow-up period (6 months vs. 4 months). Additionally, our study found a notable decrease in MDS-UPDRS scores, particularly in part IV, which assesses motor complications. These improvements were clinically meaningful [21], highlighting effective management of motor issues like wearing off and dyskinesia through interventions such as medication adjustments based on patient-recorded PD diaries, facilitating the effective management of motor complications. Moreover, the improved motor complications might be due to a significantly reduced number of non-adherence issues, as noted in a previous study [22]. These likely explain the change in scores in part IV, specifically the improvement in the time spent with dyskinesia and the OFF state. Part III scores remained unchanged. This could be because patients must consistently be in the ON state during MDS-UPDRS Part III assessments, which may explain the lack of change in Part III scores at week 24 in both groups. Parts I and II showed different changes in both groups, but these were not clinically significant [23].

Improving the QoL is a key goal of PD treatment. Several studies found no significant difference in total QoL scores after pharmacist interventions [7, 13, 24, 25], but noted improvements in specific areas like mobility [13], bodily discomfort [24], and emotional well-being [7]. Our study showing a significant decrease in overall PDQ-8 scores, including improvements in mobility, communication, bodily discomfort, and stigma subscales, following pharmaceutical care which aligns with other studies reported enhanced QoL, with reduced total PDQ scores [10, 12, 26]. In addition, non-motor symptoms like sleep disturbances, anxiety, depression, pain, gastrointestinal symptoms, and motor fluctuations significantly affect patients’ QoL [27–29]. Our study demonstrated a reduction in non-motor symptoms (e.g., sleep disturbances, pain, constipation) and OFF time in the PP group, attributed to fewer DRPs. These findings suggested pharmacist interventions can enhance PD patients’ QoL. The study employed various pharmaceutical care interventions, including medication review, DRPs assessment, adherence evaluation, patient education, counseling, shared decision-making, and telepharmacy monitoring. These interventions build trust and rapport between patients and pharmacists, ultimately improving QoL, as supported by Foppa et al. [7]. The PGIC questionnaire was used to assess overall health symptom changes, with two-thirds of patients experiencing improvement after pharmacist interventions. This aligns with other findings such as MDS-UPDRS and PDQ-8 evaluations, suggesting PGIC is better at capturing symptom changes compared to other measures like CGII [30].

### Further research

Our study utilized multifaceted interventions. Therefore, we suggest further studies should evaluate the impact of each intervention on clinical outcomes. Additionally, further research should include patients in all H&Y stages to apply the results more broadly.

## Conclusion

Our study highlights the importance of experienced pharmacists in neurology or PD clinics, collaborating with the healthcare team to enhance outcomes in PD patients. Their interventions can reduce DRPs, and improve clinical outcomes, particularly motor complications, QoL, and overall health status significantly better than usual care.

## Supplementary Information

Below is the link to the electronic supplementary material.

Supplementary file1 (DOCX 17 kb)

Supplementary file2 (DOCX 16 kb)

Supplementary file3 (DOCX 19 kb)
