# Supplementary material for: Impact of pharmacist-physician collaboration on patient outcomes in Parkinson’s disease: a randomised controlled trial in tertiary care
Source: Int J Clin Pharm. 2025 Feb 13;47(3):834–43. doi: 10.1007/s11096-025-01883-6 (PMC12125048; doi:10.1007/s11096-025-01883-6)
Supplement: Supplementary file 1 — Supplementary file1 (DOCX 17 kb) [file 11096_2025_1883_MOESM1_ESM.docx]

**Supplementary material 1:** The structured form completed by the pharmacist was reported to the physician

| **Date:** | **Time:** | **Right to health ___________________________** | | **Allergy:** □No □Yes |
| --- | --- | --- | --- | --- |
| **Case:** |  | **HN:** | **Age:** |  |
| **U/D:** |  |  |  |  |
| **Non-motor symptoms** | |  |  |  |
| 🗆 cognitive impairment  🗆 constipation  🗆 others | 🗆 hallucination  🗆 insomnia | 🗆 depressed mood  🗆 daytime sleepiness | 🗆 anxious mood  🗆 pain | 🗆 apathy  🗆 urinary problems |
| **Motor symptoms**  Onset  Duration | **Motor complications**  🗆 morning akinesia 🗆 wearing off 🗆 nocturnal hypokinesia  🗆 delay on 🗆 dyskinesia | | | |
|  | **The actual time the patient takes their meals and PD medication** | | | |
| **Food intake** | \| **1** \| **2** \| **3** \| **4** \| **5** \| **6** \| **7** \| **8** \| **9** \| **10** \| **11** \| **12** \| **13** \| **14** \| **15** \| **16** \| **17** \| **18** \| **19** \| **20** \| **21** \| **22** \| **23** \| **24** \| \| --- \| --- \| --- \| --- \| --- \| --- \| --- \| --- \| --- \| --- \| --- \| --- \| --- \| --- \| --- \| --- \| --- \| --- \| --- \| --- \| --- \| --- \| --- \| --- \| \|  \|  \|  \|  \|  \|  \|  \|  \|  \|  \|  \|  \|  \|  \|  \|  \|  \|  \|  \|  \|  \|  \|  \|  \| | | | |
| **PD medication as prescribed**  1.  2.  3.  4.  5. | \| **1** \| **2** \| **3** \| **4** \| **5** \| **6** \| **7** \| **8** \| **9** \| **10** \| **11** \| **12** \| **13** \| **14** \| **15** \| **16** \| **17** \| **18** \| **19** \| **20** \| **21** \| **22** \| **23** \| **24** \| \| --- \| --- \| --- \| --- \| --- \| --- \| --- \| --- \| --- \| --- \| --- \| --- \| --- \| --- \| --- \| --- \| --- \| --- \| --- \| --- \| --- \| --- \| --- \| --- \| \|  \|  \|  \|  \|  \|  \|  \|  \|  \|  \|  \|  \|  \|  \|  \|  \|  \|  \|  \|  \|  \|  \|  \|  \| \|  \|  \|  \|  \|  \|  \|  \|  \|  \|  \|  \|  \|  \|  \|  \|  \|  \|  \|  \|  \|  \|  \|  \|  \| \|  \|  \|  \|  \|  \|  \|  \|  \|  \|  \|  \|  \|  \|  \|  \|  \|  \|  \|  \|  \|  \|  \|  \|  \| \|  \|  \|  \|  \|  \|  \|  \|  \|  \|  \|  \|  \|  \|  \|  \|  \|  \|  \|  \|  \|  \|  \|  \|  \| \|  \|  \|  \|  \|  \|  \|  \|  \|  \|  \|  \|  \|  \|  \|  \|  \|  \|  \|  \|  \|  \|  \|  \|  \| | | | |
| \| **List of other medications** \| **Source of Medication** \| **Accuracy in medication use** \| **Note** \| \| --- \| --- \| --- \| --- \| \| 1. \|  \| □Correct □Incorrect \|  \| \| 2. \|  \| □Correct □Incorrect \|  \| \| 3. \|  \| □Correct □Incorrect \|  \| \| 4. \|  \| □Correct □Incorrect \|  \| \| 5. \|  \| □Correct □Incorrect \|  \| \| 6. \|  \| □Correct □Incorrect \|  \| \| 7. \|  \| □Correct □Incorrect \|  \| \| 8. \|  \| □Correct □Incorrect \|  \| \| 9. \|  \| □Correct □Incorrect \|  \| \| 10. \|  \| □Correct □Incorrect \|  \| \| 11. \|  \| □Correct □Incorrect \|  \| \| 12. \|  \| □Correct □Incorrect \|  \| | | | | |
| **Medication Organizer** | □Patient | □Caregiver___________________ | |  |
| **Pharmacist’s Note**   \| **DRPs** \| **Description** \| **Solutions** \| \| --- \| --- \| --- \| \| 1. \|  \|  \| \| 2. \|  \|  \| \| 3. \|  \|  \| \| 4. \|  \|  \| \| 5. \|  \|  \| | | | | |
