# Supplementary material for: Impact of pharmacist-physician collaboration on patient outcomes in Parkinson’s disease: a randomised controlled trial in tertiary care
Source: Int J Clin Pharm. 2025 Feb 13;47(3):834–43. doi: 10.1007/s11096-025-01883-6 (PMC12125048; doi:10.1007/s11096-025-01883-6)
Supplement: Supplementary file 2 — Supplementary file2 (DOCX 16 kb) [file 11096_2025_1883_MOESM2_ESM.docx]

**Supplementary material 2:** The processes of the pharmacist-physician (PP) and usual care (UC) groups

| **Pharmacist-Physician (PP) group** | **Usual Care (UC) group** |
| --- | --- |
| **Week 0**  Data collection at the clinic by the pharmacists: demographics, medical history, co-morbidities, lifestyle (such as mealtimes, bedtime, wakeup time, and types of food), laboratory findings, PD symptoms, medications, and medication adherence from medical records and face-to-face interviews | |
| **Telepharmacy-1**  Telephone calls were made by the pharmacist.  Remind them regarding their appointments, ensure they bring their medications to the clinic, and conduct a medication history to identify DRPs. | **Telepharmacy-1**  Telephone calls were made by the pharmacist.  Remind them regarding their appointments and ensure they bring their medications to the clinic. |
| **Week 12**  Both groups received usual care at the PD and Movement Disorders Clinic, led by a healthcare professional team (neurology residents, fellows, neurologists, and nurses), and obtained medications at the pharmacy department. | |
| Intervention  The pharmacist responsible for the intervention had specialized knowledge in the management of PD.  1. Prior to meeting with physicians, the pharmacist interviewed the patients/caregivers at the Parkinson’s Disease and Movement Disorders clinic as an outpatient clinic on all four aspects, following a structured form. These four aspects include: Disease Information (including the underlying disease, motor symptoms, and non-motor symptoms), Lifestyle Behaviors (mealtime, bedtime, and wake-up time), Medication Information (all current medications and how the patient uses them), and Pharmacist’s Notes (identified DRPs and proposed solutions).  2. The pharmacist documented the four aspects in the structured form as supplementary material 1, which was subsequently sent to the physicians at the outpatient Parkinson’s Disease and Movement Disorders clinic. All identified DRPs were reported to the physician through the structured form by the pharmacist.  3. Additionally, the pharmacist will propose solutions for the DRP in the pharmacist's notes section of the structured form. For DRPs requiring physician intervention, the pharmacist will collaborate with the physician to address the DRP through discussion and information sharing for joint decision-making (face-to-face conversations). However, the physician will make the final decision.  4. If the pharmacist determines that a DRP can be resolved through counseling, they will provide guidance to the patient or caregiver, document the intervention in the pharmacist’s notes section of the structured form, and notify the physician. | - |
| **Telepharmacy-2**  Telephone calls were made by the pharmacist.  Verify the medication list against the prescription to ensure completeness.  Intervention  Communication Points:  1. Explain the disease and the treatment goals.  2. Review the medication list and usage instructions (including any changes in the medication list or usage from the previous visit).  3. Inquire about and establish a specific time schedule for medication administration (providing exact times).  4. Provide precautions for medication use.  5. Provide common adverse drug reactions and provide recommendations for managing them.  6. Advise on how to avoid potential drug-drug or drug-food interactions.  7. Provide counseling on proper medication use and storage.  8. The importance of medication adherence  9. Lifestyle modifications | **Telepharmacy-2**  Telephone calls were made by the pharmacist.  Verify the medication list against the prescription to ensure completeness. |
| **Telepharmacy-3**  The same as Telepharmacy-1 | **Telepharmacy-3**  The same as Telepharmacy-1 |
| **Week 24**  Follow-up on changes in DRPs | |
