# Supplementary material for: Impact of pharmacist-physician collaboration on patient outcomes in Parkinson’s disease: a randomised controlled trial in tertiary care
Source: Int J Clin Pharm. 2025 Feb 13;47(3):834–43. doi: 10.1007/s11096-025-01883-6 (PMC12125048; doi:10.1007/s11096-025-01883-6)
Supplement: Supplementary file 3 — Supplementary file3 (DOCX 19 kb) [file 11096_2025_1883_MOESM3_ESM.docx]

**Supplementary Material 3:** Classification of drug-related problems according to Cipolle et al. and assessment of each DRP

| **DRPs** | **Assessment criteria** | **Assessment method** |
| --- | --- | --- |
| 1. Non-adherence | The following considerations:   - The patient does not take or use medication as prescribed by a physician. - The patient is self-adjusting the dosage regimen. - The patient stops taking the medication independently. - The patient does not use medication as recommended on the package insert. - The patient forgets to take the medication (if the patient indicated forgetting to take their medication at least once within a two-week period). | The pharmacist will create a detailed list of all medications the patient is currently taking before conducting the medication adherence assessment interview. The following five aspects will be assessed for all patients:  1. The pharmacist will ask about the method of administration for each medication.  2. Inquiring about mealtimes to assess whether the patient takes their medications before meals, especially levodopa.  3. Ask the patient about the specific times they take each medication, especially levodopa.  4. The pharmacist will ask the patient whether they have adjusted the dosage or stopped taking any medications on their own.  5. The pharmacist will inquire whether the patient ever forgets to take their medications. |
| 2. Adverse drug reactions | The following considerations:   - The patient experienced an undesirable effect from the medication. - The prescribed drug is unsafe for the patient. - A drug interaction causes an adverse reaction. - The patient had an allergic reaction to the medication. - The drug dosage was increased or given too quickly, causing an ADR. | The assessment was conducted using information obtained from medical records and patient/caregiver interviews. The following questions were addressed:  1. Does the patient exhibit any abnormal symptoms potentially related to medication use?  2. Are these abnormal symptoms associated with the timing of medication administration?  3. Can the abnormal symptoms be explained by other causes?  4. How does the patient manage these abnormal symptoms?  Abnormal symptoms reported by the patient were further verified using drug information databases. A joint evaluation was then performed by two pharmacists to determine whether the symptoms qualified as ADRs. |
| 3. Needs additional drug therapy | The following considerations:   - Additional drug therapy is required for prevention to reduce the risk of new medical conditions in the patient. - The patient requires additional drug therapy to address an untreated medical condition. - Additional drug therapy is needed to provide synergistic treatment for the patient's condition. | The assessment was conducted using information gathered from medical records (disease, medication list, lab results) and patient/caregiver interviews. The interview primarily focused on the patient's symptoms and medication history. The following questions were used during the interview:  1. How are your motor symptoms at the moment?  2. After taking your medication, how long does it take for your motor symptoms to improve, and how long does the improvement last? or can you describe the onset and duration of the medication’s effect that results in better mobility?  3. Do you experience involuntary movements (dyskinesia)? If so, at what time do these symptoms usually occur?  4. Do you feel stiffness, rigidity, or tremor? If so, at what time do these symptoms usually occur?  5. Can you turn over in bed at night?  6. Are you experiencing any non-motor symptoms? (as outlined in MDS-UPDRS Part I)  7. Have you taken or purchased any medications on your own (other than those listed from the hospital)?  8. Has the patient used any herbal remedies, dietary supplements, alcohol, or tobacco? |
| 4. Dosage too low | The following considerations:   - The dose is too low, resulting in an ineffective dose that fails to produce the desired response. - The dosage interval is too spaced out to produce the desired effect. - A drug interaction diminishes the effect of the drug. - The duration of treatment is too short. |  |
| 5. Dosage too high | The following considerations:   - The dose is too high. - The dosage frequency is too short. - A drug interaction may be amplifying the effects of the dosage. - The duration of treatment is too long. |  |
| 6. Unnecessary drug therapy | The following considerations:   - Unnecessary drug therapy results from duplicate therapy, in which the patient is receiving the same treatment. - There is no medical indication for the drug at this time. - The patient uses recreational drugs, alcohol, tobacco, herbs, and dietary supplements, which are unnecessary for treatment. - Drug therapy is being used to treat an avoidable adverse reaction caused by another medication, instead of addressing the root cause. |  |
| 7. Ineffective drug | The following considerations:   - The current drug is not appropriate because a more effective drug is available for the medical condition. - The drug is ineffective because the condition is refractory to it. - The dosage form is inappropriate. - Patient received a contraindicated drug. - The drug is not indicated for the patient's condition. |  |
